# Peptides derived from the knuckle epitope of BMP-9 induce the cholinergic differentiation and inactivate GSk3beta in human SH-SY5Y neuroblastoma cells

**DOI:** 10.1038/s41598-017-04835-x

**Published:** 2017-07-05

**Authors:** Marc-Antoine Lauzon, Olivier Drevelle, Nathalie Faucheux

**Affiliations:** 1 Department of Chemical and Biotechnological Engineering, Université de Sherbrooke, 2500 boul. de l’Université, Sherbrooke, Québec J1K 2R1 Canada; 2 Department of Chemical Engineering, École Polytechnique de Montréal, 2900, Blvd Édouard Montpetit, Montréal, Québec H3C 3A7 Canada; 3 Clinical Research Center of Centre Hospitalier Universitaire de Sherbrooke, 12e Avenue N, Sherbrooke, Québec J1H 5N4 Canada

## Abstract

The incidence of brain degenerative disorders like Alzheimer’s disease (AD) will increase as the world population ages. While there is presently no known cure for AD and current treatments having only a transient effect, an increasing number of publications indicate that growth factors (GF) may be used to treat AD. GFs like the bone morphogenetic proteins (BMPs), especially BMP-9, affect many aspects of AD. However, BMP-9 is a big protein that cannot readily cross the blood-brain barrier. We have therefore studied the effects of two small peptides derived from BMP-9 (pBMP-9 and SpBMP-9). We investigated their capacity to differentiate SH-SY5Y human neuroblastoma cells into neurons with or without retinoic acid (RA). Both peptides induced Smad 1/5 phosphorylation and their nuclear translocation. They increased the number and length of neurites and the expression of neuronal markers MAP-2, NeuN and NSE better than did BMP-9. They also promoted differentiation to the cholinergic phenotype more actively than BMP-9, SpBMP-9 being the most effective as shown by increases in intracellular acetylcholine, ChAT and VAchT. Finally, both peptides activated the PI3K/Akt pathway and inhibited GSK3beta, a current AD therapeutic target. BMP-9-derived peptides, especially SpBMP-9, with or without RA, are promising molecules that warrant further investigation.

## Introduction

Alzheimer’s disease (AD) is the most common type of dementia, accounting for about 60% of all cases, affecting over 40 million people worldwide^1^. However, there is no cure for AD and the therapies currently available or under investigation have only transient effects and slow disease progression^2, 3^. Most target only one of the three major hallmarks of AD at a time (cholinergic system dysfunction^4^, beta amyloid plaque accumulation^5^ and Tau protein hyperphosphorylation^6, 7^), although considerable evidence suggests that these hallmarks are all intimately linked^8–10^. Growth factors (GFs) like neurotrophins (nerve growth factor and brain-derived neurotrophic factor), bone morphogenetic proteins (BMPs) and insulin-like growth factor 2 (IGF-2), which are found in the developing and healthy mature brain, but are dysregulated in AD, seem to prevent the evolution of the disease. They could act on several AD hallmarks simultaneously and repair the dysfunctional cell signalling^8, 11–16^.

One subfamily of GFs, the BMPs, may have great potential as they are involved in brain development, maintenance and homeostasis^8, 17–19^. The BMPs, more than 20 at the last count, were discovered in bone tissue by Urist and Strates in the early 1970s^20–22^. BMPs signal in the brain via their type I and type II Serine/Threonine kinase receptors and activate the canonical Smad pathway (Smad 1/5/8), which is important in early brain development and neuron maturation^19, 23, 24^.

One BMP, BMP-9, may be a promising candidate for therapy: it is present in the brain and seems to be linked to the function of cholinergic neurons^25, 26^. Lopez-Coviella *et al*. found high amount of BMP-9 transcripts in the spinal cord and septal area of mice^11^. BMP-9 also activates the transcriptome of basal forebrain cholinergic neurons, which are associated with learning and memory, and is directly implicated in their differentiation^25^. Other evidence indicates that BMP-9 has a direct therapeutic effect on AD hallmarks^11–13, 26^. Lopez-Coviella *et al*. studied mouse medial septum neurons and showed that BMP-9 increases the expression of cholinergic markers in the hippocampus and prevents the loss of choline acetyltransferase (ChAT); this loss is characteristic of AD-associated memory loss^11^. Similarly, Burke *et al*., working on a mouse model of AD (APP.PS1, transgenic mouse overproducing β–amyloid peptides), demonstrated that intraventricular injections of BMP-9 dramatically reduced the accumulation of senile plaques in the cortex and hippocampus, and increased the ChAT levels^13^.

But despite its interesting therapeutic features, recombinant human BMP-9 is expensive to produce and is not readily introduced into the brain by systemic or nasal injection because of its size (homodimer of 24 kDa) and the great selectivity of the blood brain barrier. Also, BMPs have short half-lives in the circulatory system^27^. We have therefore developed small (23 residues) and relatively inexpensive (300-times less expensive than BMP-9) peptides derived from the knuckle epitope of BMP-9. They correspond to the sequence recognized by the BMP type II receptor (BMPRII) and are based on the work of Suzuki *et al*.^28^ and Saito *et al*.^29^ on peptides derived from BMP-2. These peptides trigger signalling and cell responses similar to those induced by the whole protein (BMP-9) in the context of bone regeneration^30–32^ but their effects on neurons remain unknown. We therefore explored the effects of two BMP-9-derived peptides (pBMP-9^30^ and SpBMP-9^32^) on SH-SY5Y human neuroblastoma cells, a well-known human mature neuron cell model that has been used several times in the context of AD^33–35^. Recombinant human BMP-9 was used as a control in all experimental conditions. We also stimulated SH-SY5Y cells in the presence of retinoic acid (RA), a potent agent of neuron differentiation^36^ that is believed to be therapeutic for AD^37^.

## Results

### pBMP-9 and SpBMP-9 activate the Smad1/5 pathway

#### Effect of pBMP-9 and SpBMP-9 on kinetic of Smad 1/5 phosphorylation

**Figure 1:**
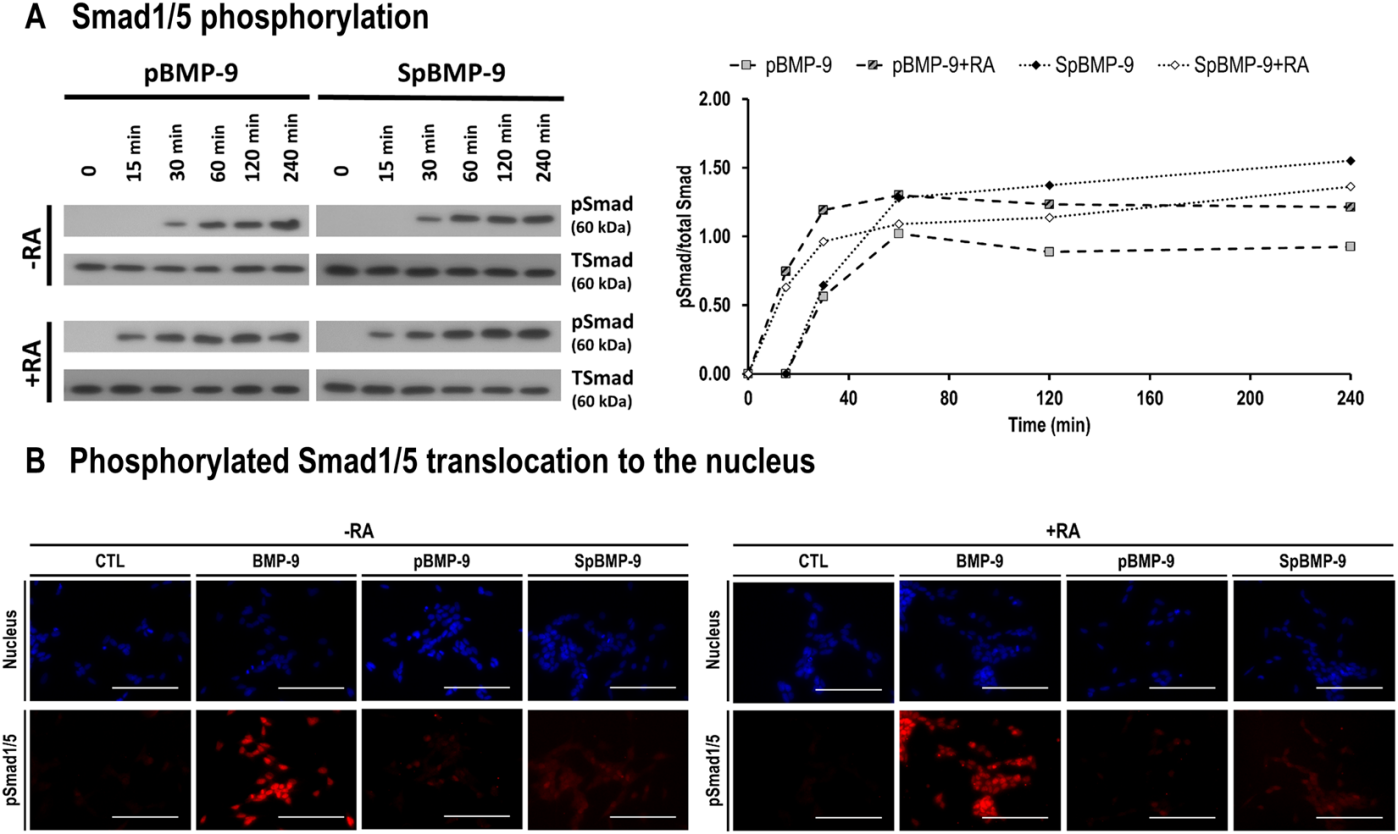
Effect of pBMP-9 and SpBMP-9 on the activation of the canonical Smad1/5 pathway. (**A**) Western blots of phosphorylated Smad1/5 (pSmad) and densitometric analysis of pSmad1/5 as referred to total Smad (TSmad) showing the effect of 1 nM BMP-9, pBMP-9 and SpBMP-9 +/− 10 μM RA in SH-SY5Y cells after incubation for 0, 15, 30, 60, 120 and 240 min. (**B**) Pictures showing the nucleus (blue) and the pSmad1/5 (red) in SH-SY5Y cells stimulated with an equimolar concentration of BMP-9, pBMP-9 or SpBMP-9 (1 nM) +/− 10 μM RA after incubation for 240 min. Results are representative of 2 independent experiments performed in duplicate (Bar = 100 μm).

We have recently shown that BMP-9 (1 nM) with or without RA can activate the canonical Smad1/5 pathway within 30 min in SH-SY5Y cells^8^. We therefore verified the ability of both pBMP-9 and SpBMP-9 (1 nM) to activate the Smad 1/5 pathway (Fig. 1A). The total Smad1/5 (TSmad) was used as a control. Without RA, the phosphorylated Smad1/5 (pSmad) bands were detected after incubation for 30 min in cells stimulated with pBMP-9 or SpBMP-9. Both peptides in the presence of RA induced the phosphorylation of Smad1/5 at 15 min. The Smad1/5 remained phosphorylated within 240 min in the presence of pBMP-9 or SpBMP-9 with or without RA. Densitometric analysis of bands corresponding to pSmad1/5 and standardized to that of TSmad (Fig. 1A) confirmed that pSmad1/5 levels reached a plateau after 1 h.

#### Effect of pBMP-9 and SpBMP-9 on the nuclear translocation of phosphorylated Smad1/5

Since pBMP-9 and SpBMP-9 induced the phosphorylation of Smad 1/5 (pSmad1/5) in SH-SY5Y cells, we then analyzed the pSmad1/5 translocation into the nucleus after incubation for 240 min by labelling both DNA (nucleus) and pSmad1/5 (Fig. 1B). Without RA, cells incubated with BMP-9 (1 nM) had a strong pSmad1/5 staining compared to untreated cells (CTL) and the pSmad1/5 were mainly located at the nucleus. Both pBMP-9 and SpBMP-9 at 1 nM induced a slight pSmad1/5 labelling compared to BMP-9. Only some cells possessed a strong nuclear translocation of the pSmad1/5. The same tendencies were observed in cells treated by BMP-9 or its derived peptides in the presence of RA.

### pBMP-9 and SpBMP-9 do not affect the number of SH-SY5Y cells

#### Effect of pBMP-9 and SpBMP-9 on cell metabolic activity and viability

**Figure 2:**
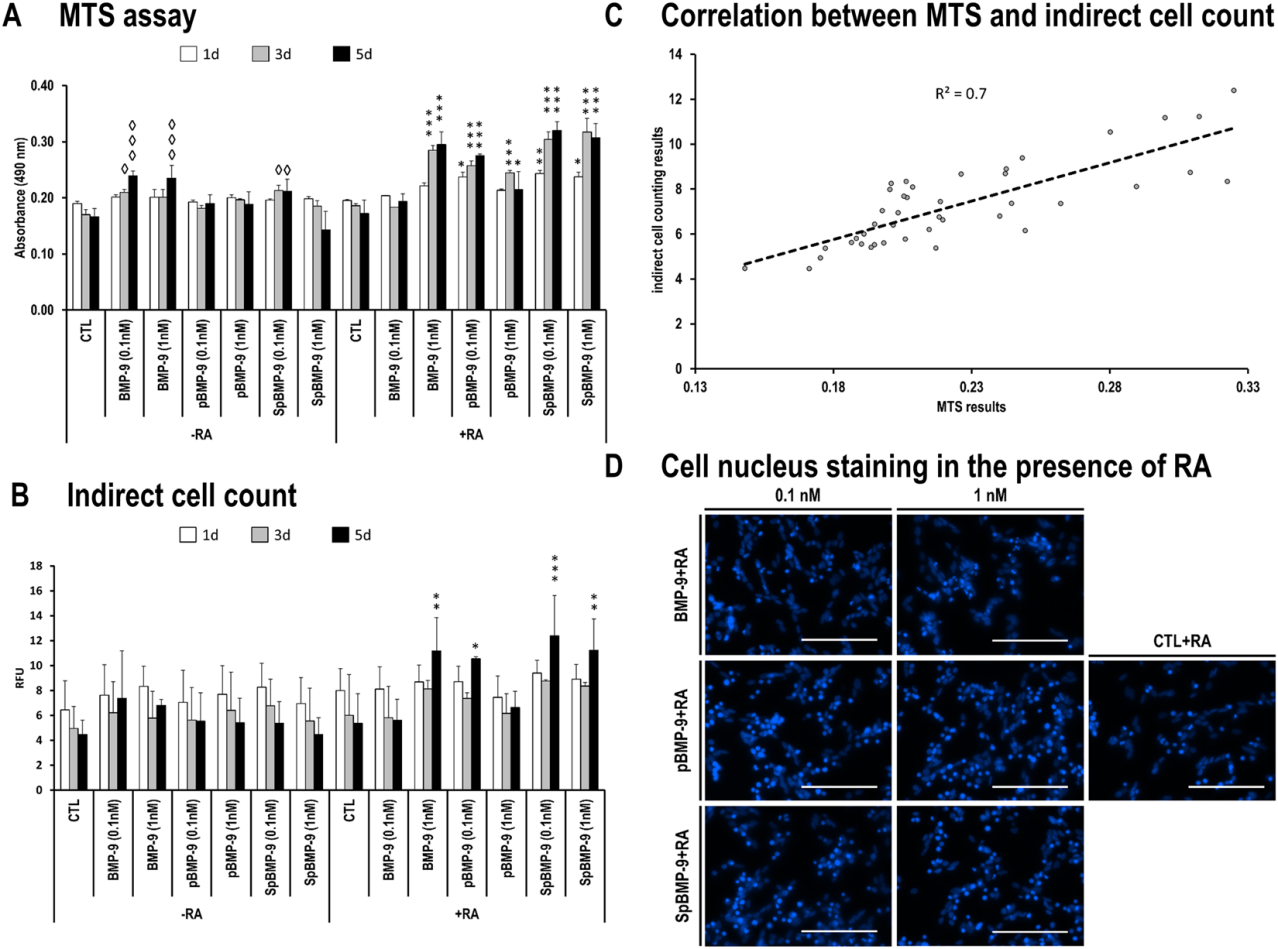
Effect of pBMP-9 and SpBMP-9 on the metabolic enzymatic activity and indirect cell counting. (**A**) MTS assays of SH-SY5Y cells stimulated for 1d, 3d and 5d with equimolar concentrations (0.1 and 1 nM) of BMP-9, pBMP-9 and SpBMP-9 +/− 10 μM RA in serum-free culture medium (⬨p < 0.05 and ⬨⬨⬨p < 0.001 compared with the control without RA, *p < 0.05, **p < 0.01 and ***p < 0.001 compared with the control with RA). Results are the means ± SEM of at least two independent experiments performed in duplicate. (**B**) Indirect cell counting showing the relative fluorescence intensity of the stained nuclei in cells used to perform the MTS assays (*p < 0.05, ** p < 0.01, ***p < 0.001). (**C**) Indirect cell counting results plot in function of the MTS enzymatic assay results showing a correlation of 0.7. (**D**) Cell nucleus staining in SH-SY5Y cells stimulated by BMP-9, pBMP-9 or SpBMP-9 (0.1 and 1 nM) in the presence of 10 μM RA for 5d (Bar = 100 μm).

We investigated the influence of pBMP-9 and SpBMP-9 with or without RA on the viability of SH-SY5Y cells. BMP-9 was used as a control. We measured the activity of the enzyme succinate dehydrogenase using the MTS assay (Fig. 2A). The control (CTL) showed no alteration in the metabolic activity either with or without RA. Incubation for 3d with 0.1 nM BMP-9 or 0.1 nM SpBMP-9 but no RA resulted in a significant increase in metabolic activity (p < 0.05) in comparison to the CTL. Similar results were obtained after incubation for 5d with 0.1 or 1 nM BMP-9 and with 0.1 nM SpBMP-9. Cells incubated with 0.1 or 1 nM BMP-9 and its derived peptides plus RA for 3d and 5d had significantly increased the metabolic activity.

#### Effect of pBMP-9 and SpBMP-9 on cell number

Cell nucleus was stained with Hoechst 33342 to verify whether the observed increase in metabolic activity were caused by increases in cell numbers (Fig. 2B). In the absence of RA, there was no significant difference between experimental conditions, neither in function of time. In the presence of RA, after 5d of incubation, cells stimulated with BMP-9 (1 nM), pBMP-9 (0.1 nM) or SpBMP-9 (0.1 nM) had a significant increased amount of nucleus relative fluorescence intensity in comparison with the control, those results being in accordance with the metabolic enzymatic assay. There was a determination coefficient of 0.7 between indirect cell counting and MTS assay results, which indicates that the metabolic activity might be related to the number of cells (Fig. 2C).

However, since the cell number was evaluated indirectly by measuring the relative fluorescence intensity of the nucleus staining, we then looked at the cell nucleus in a higher magnification to evaluate if the increase in the cell number in experiments performed with RA could rather be explained by the presence of pyknotic cell nuclei (Fig. 2D). There were some pyknotic nuclei for cells stimulated for 5d in the presence of BMP-9, pBMP-9 and SpBMP-9 plus RA. From those results, it appears that cells stimulated with BMP-9, pBMP-9 and SpBMP-9, especially in the presence of RA increase their metabolic activity, which is not caused by an increase in cell number.

### pBMP-9 and SpBMP-9 affect the morphology of SH-SY5Y cells and increase their neurites outgrowth

#### Effect of pBMP-9 and SpBMP-9 on cell morphology

**Figure 3:**
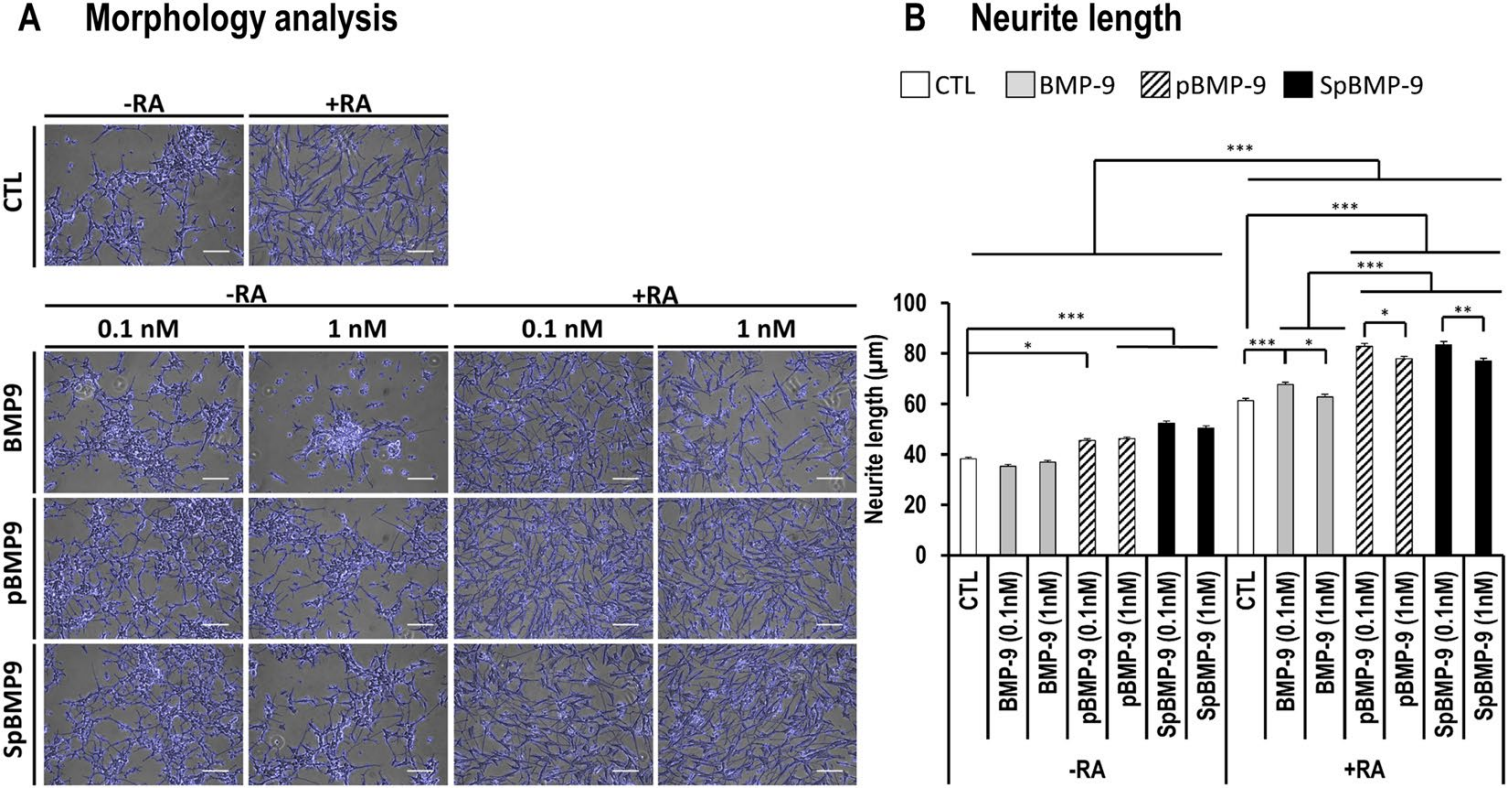
Effect of pBMP-9 and SpBMP-9 on the morphology of SH-SY5Y cells and neurite length. (**A**) Modified phase-contrast pictures of SH-SY5Y cells stimulated for 5d with equimolar concentrations (0, 0.1 and 1 nM) of BMP-9, pBMP-9 and SpBMP-9 +/− 10 μM RA. Pictures are representative of at least 3 independent experiments performed in duplicate. Neurites and cell bodies were highlighted with an edge-detection filter and superimposed (blue edges) on the original phase contrast image (Bar = 100 μm). (**B**) Average neurite length of SH-SY5Y cells stimulated for 5d with equimolar concentration of BMP-9, pBMP-9 or SpBMP-9 (0.1 and 1 nM) +/− 10 μM RA determined as the Euclidean distance between the end of neurites and the cell body. Results are the means ± SEM of at least 3 independent experiments performed in duplicate, where a total of over 300 measurements were taken per experimental condition (*p < 0.05, **p < 0.01, ***p < 0.001).

The impact of BMP-9, pBMP-9 and SpBMP-9 on neuron well-being and the differentiation of cells like SH-SY5Y require an examination of the cell morphology (Fig. 3A). Cells incubated in serum free-medium with BMP-9, especially at 1 nM, reacted more like neuroblastoma cancerous cells, forming clusters with less neuron-like morphology. Cells stimulated with pBMP-9 and SpBMP-9 show an increase amount of neurite outgrowth. RA had a dramatic effect on cell morphology under all experimental conditions: it increased neurite outgrowth and displayed more neuronal morphology. Cells stimulated with pBMP-9 or pBMP-9 plus RA had more interconnected neurites than did control plus RA.

We then measured the average length of neurites under each experimental condition (Fig. 3B). SH-SY5Y cells stimulated with pBMP-9 (at least p < 0.01) or SpBMP-9 (p < 0.001) without RA had longer neurites than those stimulated with BMP-9 or the CTL. Adding RA increased the neurite length under all experimental conditions. Cells stimulated with 0.1 nM BMP-9 or 0.1 and 1 nM pBMP-9 and SpBMP-9 plus RA had significant (p < 0.001) longer neurites than the corresponding CTL, whereas the neurites of cells stimulated with 1 nM BMP-9 plus RA were not significant different from those of CTL. The lower concentrations (0.1 nM) of both pBMP-9 (p < 0.05) and SpBMP-9 (p < 0.01) stimulated the formation of significantly longer neurites than did the higher concentration (1 nM).

### pBMP-9 and SpBMP-9 increase the expression of early and late neuronal differentiation markers

#### Effect of stimulation time on neuron markers expression

**Figure 4:**
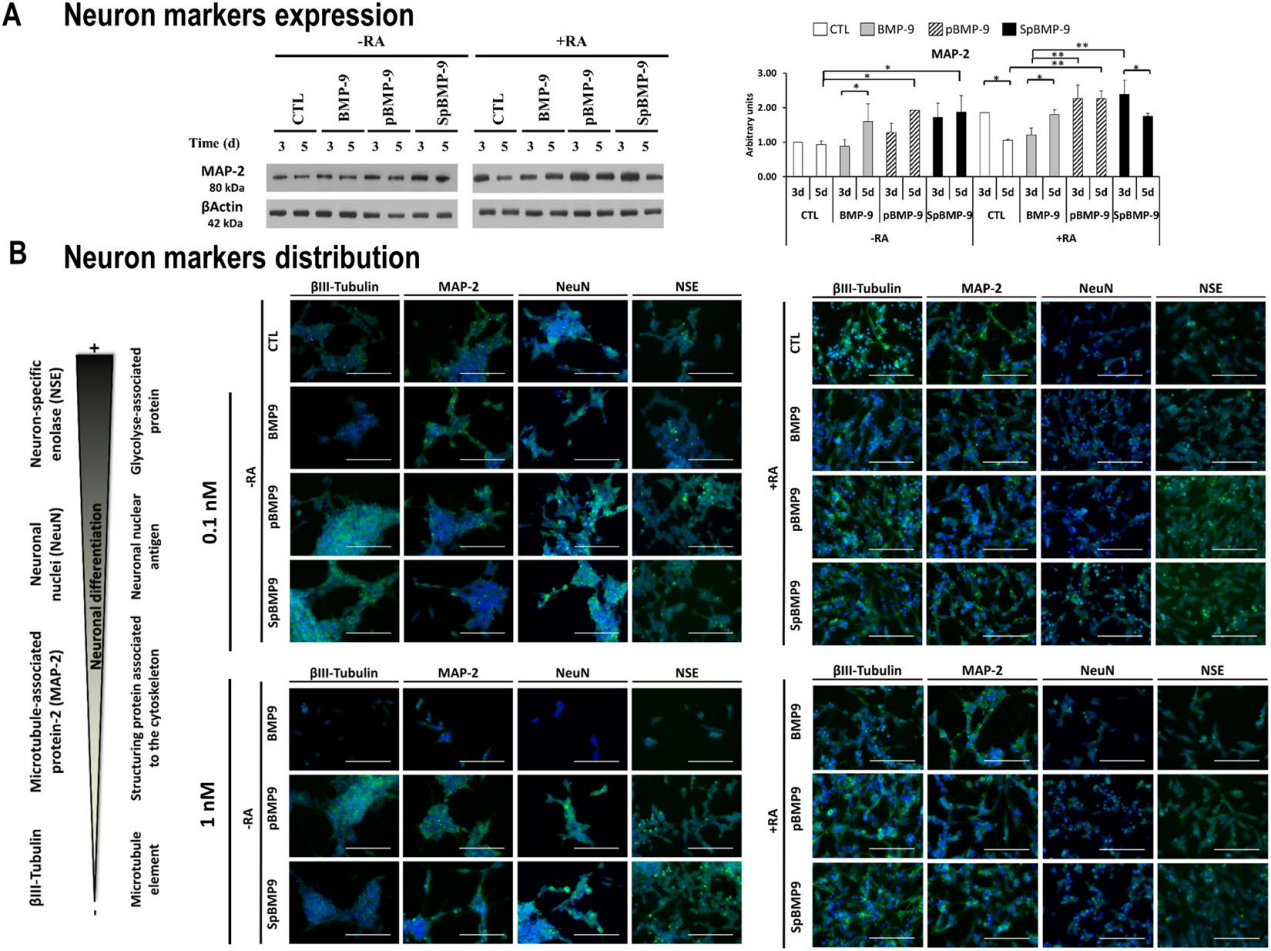
Effect of pBMP-9 and SpBMP-9 on the differentiation of SH-SY5Y cells. (**A**) Western blots of MAP-2 and βactin (2 independent experiments) and densitometric analyses of MAP-2 bands normalized to βactin (means ± SEM) for SH-SY5Y cells stimulated with 0.1 nM BMP-9, pBMP-9 and SpBMP-9 +/− 10 μM RA for 3d and 5d (*p < 0.05, **p < 0.01). Only cropped pictures of western blots showing the 80 kDa MAP-2 isoform were presented in order to allow a better comparison between experimental conditions. Complete gel pictures are available in the supplementary data file. (**B**) Merged pictures showing immunostaining for neuronal differentiation markers βIII-tubulin (Alexa Fluor® 488, green), MAP-2 (Alexa Fluor® 488, green), NeuN (FITC, green) and NSE (FITC, green), and nuclei staining (Hoechst, blue) of SH-SY5Y cells stimulated for 5d with 0, 0.1 or 1 nM BMP-9, pBMP-9 and SpBMP-9 +/− 10 μM RA. Pictures are representative of at least two independent experiments (Bar = 100 μm).

Since pBMP-9 and SpBMP-9 had an effect on the morphology of SH-SY5Y cells inducing neurite formation characteristic of the neuron phenotype, we used Western blots to evaluate the expression of MAP-2 protein, an early marker of neuron differentiation^38^ (Fig. 4A). There was significantly more of MAP-2 in cells stimulated with pBMP-9 and SpBMP-9 (0.1 nM) without RA for 5d than in the CTL as shown by the densitometric analyses (p < 0.05). There was a significant increase in MAP-2 in cells stimulated for 3d with pBMP-9 or SpBMP-9 plus RA in comparison to BMP-9 plus RA (p < 0.01). Finally, the expression of MAP-2 decreased between 3d and 5d in the CTL (p < 0.05) and in cells treated with SpBMP-9 (p < 0.05), while it increased in cells stimulated with BMP-9 (p < 0.05). The MAP-2 levels in cells stimulated with pBMP-9 for 3d and 5d were the same.

#### Neuron markers distribution

Knowledge of the distribution of neuron differentiation markers in cells is essential for confirming the neuron phenotype. For example, NeuN is specific to neuron cells and is restricted to the cell nucleus^39, 40^. We used immunolabelling to determine the effect of BMP-9, pBMP-9 and SpBMP-9 on the expression and distributions of the markers of early (βIII-tubulin, MAP-2) and late neuron differentiation (NeuN and NSE) in cells incubated for 5d (Fig. 4B).

We detected βIII-tubulin under all experimental conditions, but it was most prominent in cells stimulated with RA for all experimental conditions. BMP-9 without RA produced less intense labelling regardless of its concentration. However the cells stimulated with pBMP-9 or SpBMP-9 contained more markers than did the control or those stimulated with BMP-9 with or without RA.

MAP-2 was most abundant in the neurites of SH-SY5Y cells stimulated with 0.1 or 1 nM pBMP-9 or SpBMP-9, especially in the presence of RA. Since MAP-2 is a microtubule-associated protein, an increase in its expression and its distribution in the neurites is associated with greater neuronal differentiation^38^. Staining for the marker of late differentiation, NeuN, in cell nuclei^39^, was more intense in cells stimulated with pBMP-9, and especially SpBMP-9, with or without RA, than in cells stimulated with BMP-9 or the CTL (Fig. 4B). SH-SY5Y cells stimulated with 1 nM BMP-9 were poorly stained, especially those incubated without RA. However, there was some staining in the cell body around the nucleus. This could be due to the presence of synapsin I, which is recognized by NeuN antibodies^41^. Finally, incubation with pBMP-9 or SpBMP-9 produced the best staining for NSE, a late marker located in the cell cytoplasm, regardless of their concentration or the presence of RA.

These results indicate that pBMP-9 and SpBMP-9 induce more rapid neuron differentiation than the whole BMP-9 protein or the CTL, especially in the presence of RA. However, greater neuron differentiation does not necessarily mean that cells are moving toward the cholinergic phenotype.

### SpBMP-9 increases the expression of choline acetyltransferase, vesicular acetylcholine transporter protein and the level of intracellular acetylcholine

**Figure 5:**
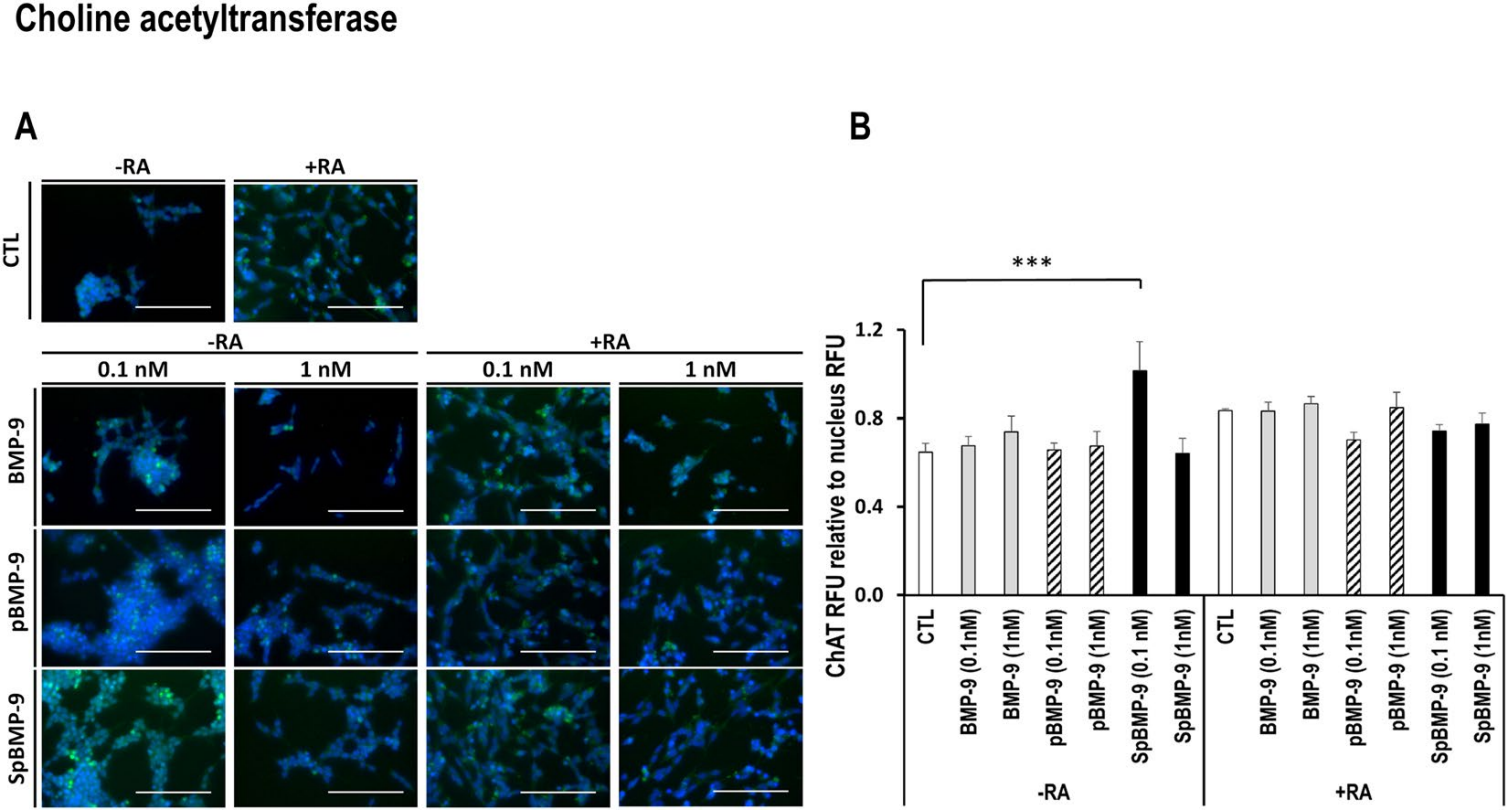
Effect of pBMP-9 and SpBMP-9 on the expression of choline acetyltransferase. (**A**) Merged pictures showing immunostaining for ChAT (FITC, green) and nuclei labelling (Hoechst, blue) of SH-SY5Y cells stimulated for 5d with 0, 0.1, or 1 nM BMP-9, pBMP-9 and SpBMP-9 +/− 10 μM RA (Bar = 100 μm). Pictures are representative of at least 2 independent experiments. (**B**) Analysis of ChAT fluorescence intensity relative to the nucleus staining was also presented. Results are the means ± SEM (***p < 0.001).

**Figure 6:**
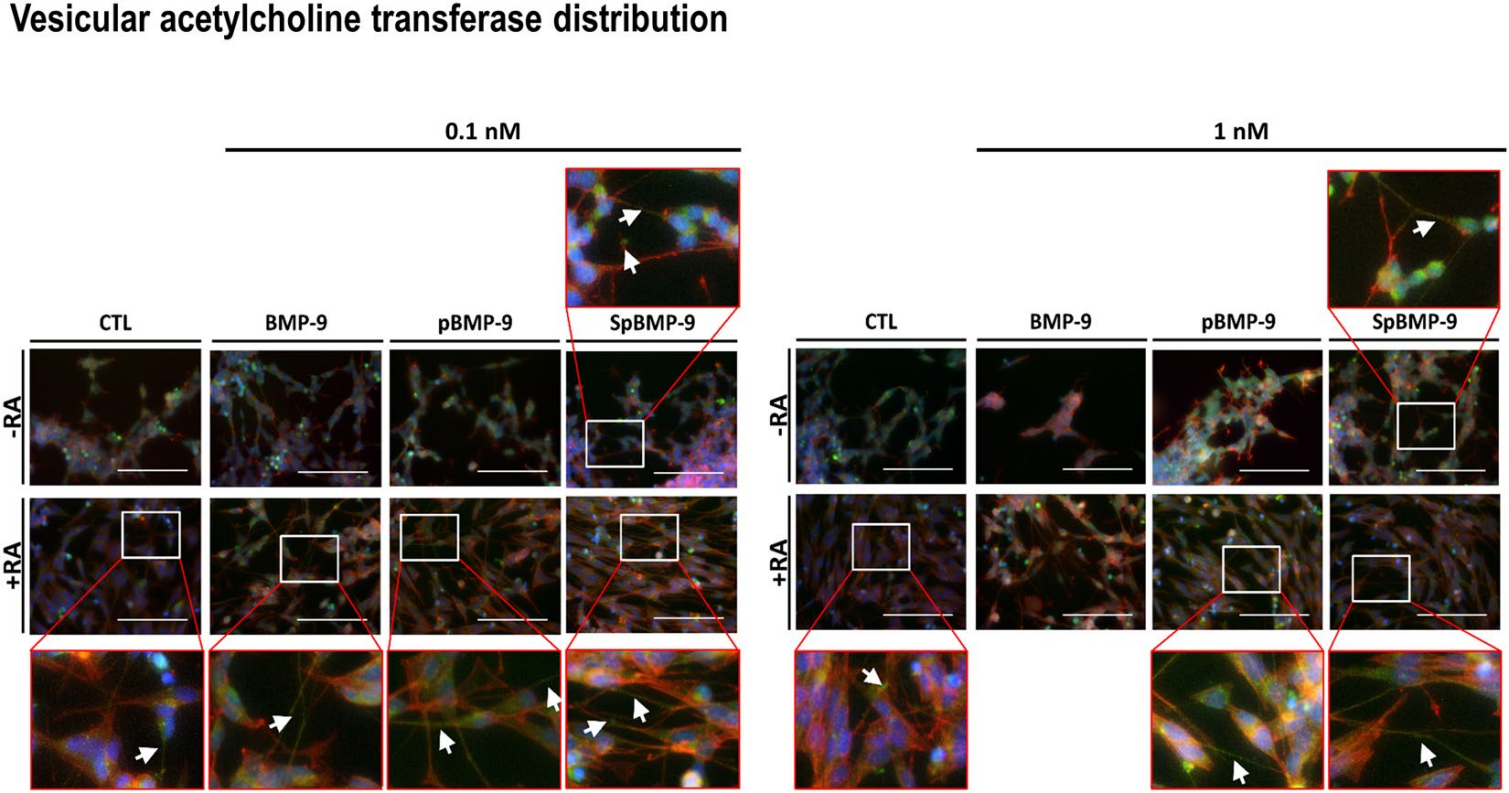
Effect of pBMP-9 and SpBMP-9 on the expression and the distribution of VAchT. Merged pictures showing immunostaining for VAchT (FITC, green), actin cytoskeleton (rhodamine-phalloidin, red) and nuclei labelling (Hoechst, blue) of SH-SY5Y cells stimulated for 5d with 0, 0.1 or 1 nM BMP-9, pBMP-9 and SpBMP-9 +/− 10 μM RA. White squares show magnified zones, white arrows indicate VAchT vesicles in cell neurites (Bar = 100 μm). Pictures are representative of at least 2 independent experiments.

**Figure 7:**
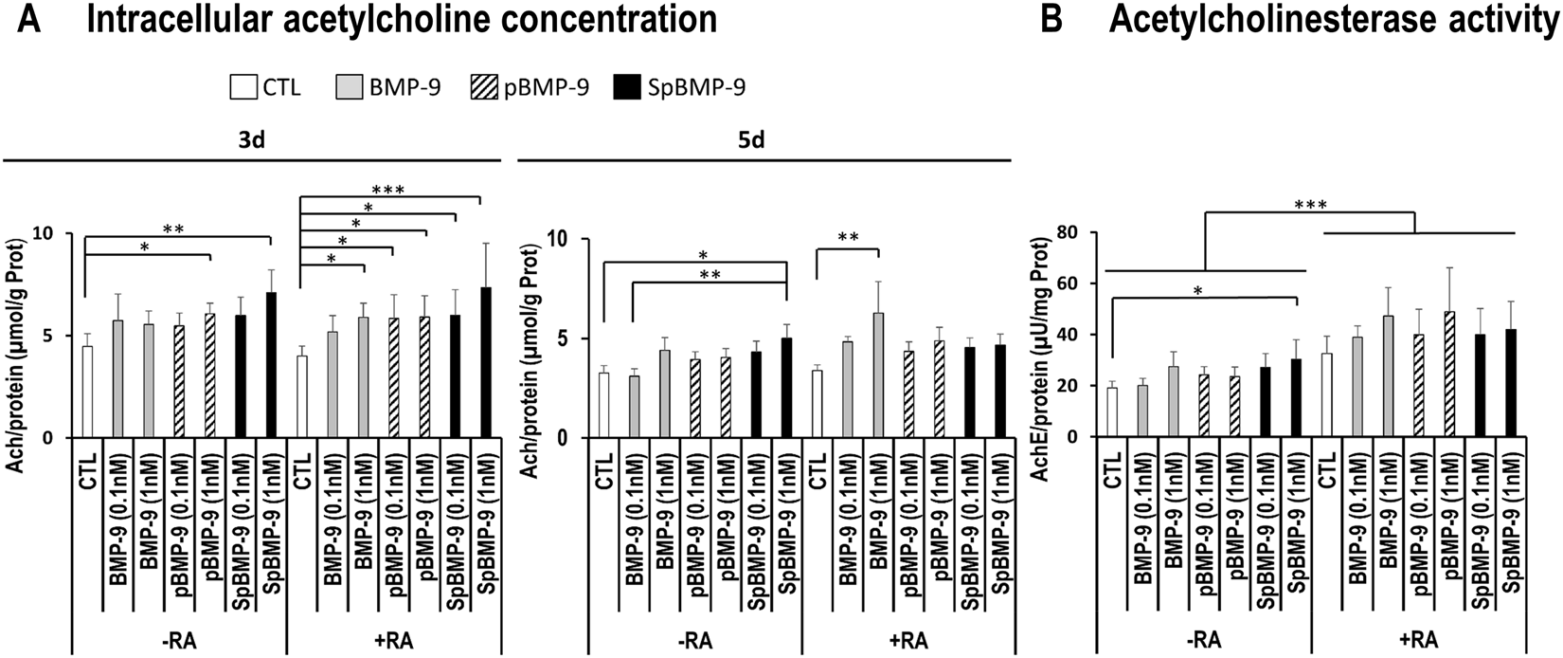
Effect of pBMP-9 and SpBMP-9 on the intracellular Ach and AchE. (**A**) Intracellular Ach in SH-SY5Y cells stimulated with 0, 0.1 or 1 nM BMP-9, pBMP-9 and SpBMP-9 +/− 10 μM RA for 3d and 5d (**B)** AchE activity for SH-SY5Y cells stimulated with 0, 0.1 or 1 nM BMP-9, pBMP-9 and SpBMP-9 +/− 10 μM RA for 5d. Results are the means ± SEM of at least 4 independent experiments (*p < 0.05, **p < 0.01, ***p < 0.001).

BMP-9 activates the cholinergic transcriptome of murine septal cells and basal forebrain cholinergic neurons and increase the levels of Ach and the vesicular acetylcholine transporter protein (VAchT)^11, 25, 26^. Ach is synthesized in the cell body by fusion of acetyl Co-A and choline catalysed by choline acetyltransferase (ChAT); it is then transported to vesicles by VAchT. The *ChAT* and *VAchT* genes are conserved at the same locus, which suggests that their expressions are coordinated^42^. We investigated the effect of pBMP-9 and SpBMP-9 on the induction and the maintenance of the cholinergic phenotype since cholinergic dysfunction is a major hallmark of AD (Figs 5–7).

#### Effect of pBMP-9 and SpBMP-9 on choline acetyltransferase

The ChAT enzyme responsible for converting acetyl-co-A and choline to acetylcholine in SH-SY5Y cells incubated with BMP-9 or its derived peptides with or without RA was detected by immunolabelling (Fig. 5A and B). We found ChAT immunostaining in all cell bodies under all experimental conditions (Fig. 5A). However, the intensity of labelling differed, especially when the cells were stimulated with SpBMP-9 without RA. The CTL without RA had the lowest ChAT staining, while cells incubated with 0.1 nM SpBMP-9 had the highest ones as confirmed by the relative fluorescence intensity analysis (p < 0.05) (Fig. 5B). Cells stimulated with BMP-9 and pBMP-9 had similar fluorescence intensities as the control, which were lower than that of SpBMP-9-stimulated cells. However, no difference in relative fluorescence intensities was observed when the same assays were run in the presence of RA.

#### Effect of incubation time and pBMP-9 or SpBMP-9 dose on intracellular acetylcholine concentration and VAchT expression and distributions within cells

VAchT in the axon terminals plays an important role in the accumulation of Ach prior to its release^43^. We immunolabelled VAchT to evaluate the effects of SpBMP-9 and pBMP-9 on its expression and distribution in the cells (5d, Fig. 6). The presence of labelled VAchT in small vesicles within the neurites indicates a cholinergic differentiation. Only cells stimulated with 0.1 nM or 1 nM SpBMP-9 (without RA) had VAchT vesicles in their neurites, with 1 nM being more efficient. RA alone significantly stimulated VAchT accumulation in the neurites. Cells stimulated with 0.1 nM BMP-9, 0.1 and 1 nM pBMP-9 and SpBMP-9 plus RA all had similar amounts and distributions of VAchT vesicles in the neurites. However, cells stimulated with 1 nM BMP-9 contained no vesicles.

We then measured intracellular Ach to determine the abilities of BMP-9 and its derived peptides to stimulate the synthesis of Ach in SH-SY5Y cells (Fig. 7A). Only 1 nM SpBMP-9 (p < 0.01) and pBMP-9 (p < 0.05) significantly stimulated Ach synthesis after incubation for 3d without RA. The concentration of Ach in SH-SY5Y cells stimulated with 1 nM SpBMP-9 for 5d was also higher than that in unstimulated cells (p < 0.05) or cells stimulated with 0.1 nM BMP-9 (p < 0.01). Cells incubated for 3d in the presence of RA plus 1 nM BMP-9, 0.1 or 1 nM pBMP-9 or SpBMP-9 contained more Ach than did the CTL, while those incubated with SpBMP-9 had the highest Ach concentration (p < 0.001). For the SH-SY5Y cells stimulated with BMP-9, pBMP-9 or SpBMP-9 for 5d in the presence of RA, intracellular Ach levels, except for cells stimulated with 1 nM of BMP-9, were similar to the CTL plus RA.

#### Effect of pBMP-9 and SpBMP-9 on acetylcholinesterase activity

We measured AchE activity to determine the action of SpBMP-9 on Ach breakdown (Fig. 7B). AchE cleaves the neurotransmitter released into the synaptic clef to give choline and acetic acid; the choline is then recycled back into the cells. Only the SH-SY5Y cells incubated for 5d with SpBMP-9, but no RA, contained AchE activity that was significantly enhanced in comparison to the CTL (p < 0.05) (Fig. 7B). However, the presence of RA increase significantly in all experimental conditions the relative AchE activity compared to the cells without RA (p < 0.001). In addition, cells incubated with BMP-9 or its derived peptides plus RA had AchE activities similar to the control plus RA.

### pBMP-9 and SpBMP-9 increase the activation PI3K/Akt pathway and inactivate GSK3β

Since SpBMP-9 has been shown to induce a higher cholinergic differentiation of SH-SY5Y cells, we then evaluated the effect of SpBMP-9 on the activation state of GSK3β, a Tau kinase^44^. As GSK3β can be inactivated by Akt-catalysed phosphorylation of its Ser9^44, 45^, we first analyzed the activation state of Akt in cells incubated with BMP-9, pBMP-9 or SpBMP-9 with and without RA.

#### Phosphorylation of Akt at Thr308

**Figure 8:**
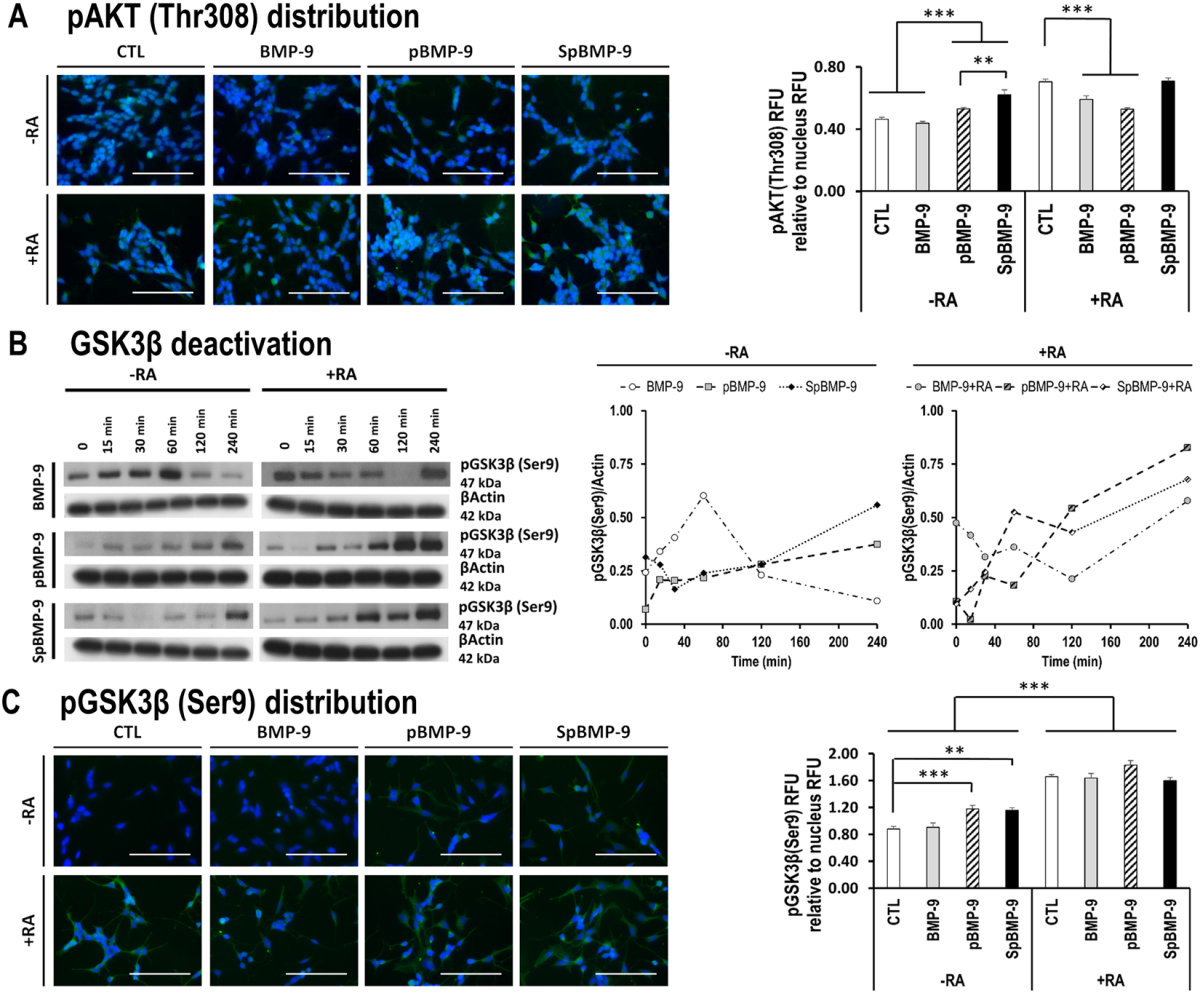
Effect of pBMP-9 and SpBMP-9 on the PI3K/Akt/GSK3β pathway. (**A**) Merged pictures representative of at least 2 independent experiments showing immunostaining for pAkt (Thr308) (green) and nuclei (Hoechst, blue) of SH-SY5Y cells stimulated for 2 h with 0.1 nM BMP-9, pBMP-9 and SpBMP-9 +/− 10 μM RA (Bar = 100 μm) and pAkt(Thr308) fluorescence activity relative to the nucleus. (**B)** Western blots of phosphorylated GSK3β at Ser9 (pGSK3β) and densitometric analysis of pGSK3β bands standardized by actin showing the effect of 0.1 nM BMP-9, pBMP-9 and SpBMP-9 +/− 10 μM RA in SH-SY5Y cells after incubation for 0, 15, 30, 60, 120 and 240 min. Only cropped pictures of western blots were shown in order to allow a better comparison between experimental conditions. Complete gel pictures are available in the supplementary data file. (**C)** Merged pictures representative of at least 2 independent experiments showing immunostaining for pGSK3β (Ser9) (FITC, green) and nuclei (Hoechst, blue) in SH-SY5Y cells stimulated for 4 h with 0.1 nM BMP-9, pBMP-9 and SpBMP-9 +/− 10 μM RA (Bar = 100 μm). Analysis of pGSK3β(Ser9) fluorescence intensity relative to the nucleus staining was also presented. Results are the means ± SEM (**p < 0.01, ***p < 0.001).

We analyzed the effect of equimolar concentration of BMP-9, pBMP-9 or SpBMP-9 (0.1 nM) with or without RA on the phosphorylation of Akt at its catalytic site (Thr308) by immunostaining (Fig. 8A). Cells stimulated with pBMP-9 or SpBMP-9 showed a higher level of fluorescence corresponding to pAkt (Thr308) compared to unstimulated cells or those incubated with BMP-9, as confirmed by relative fluorescence intensity analyses of these immunolabellings (p < 0.001). SpBMP-9 was also most effective than pBMP-9 (p < 0.01). In the presence of RA, Akt was phosphorylated at Thr308 in all experimental conditions. BMP-9 or pBMP-9 plus RA induced slightly less pAkt on Thr308 in comparison to RA alone (p < 0.001).

#### Inactivation of GSK3β by its phosphorylation at Ser9

We used Western blotting to assess phosphorylated GSK3β (pGSK3β) on Ser9 to determine whether Akt activation leads to inactivation of GSK3β (Fig. 8B). BMP-9, without RA, had a transient effect as confirmed by densitometric analysis of bands corresponding to pGSK3β and standardized to that of β actin: pGSK3β (Ser9) increased between 0 and 60 min, plateaued and then decreased from 120 to 240 min. Incubation with pBMP-9 and SpBMP-9 gave a different activation pattern. The pGSK3β (Ser9) in cells incubated with either pBMP-9 or SpBMP-9 increased from 0 to 240 min. Cells incubated with pBMP-9 or SpBMP-9 plus RA had similar time-dependent increases in pGSK3β (Ser9). pGSK3β (Ser9) was detected after 240 min in cells incubated with BMP-9, pBMP-9 or SpBMP-9 plus RA. We confirmed these observations by immunostaining for pGSK3β (Ser9) at 4 h (Fig. 8C). pBMP-9 and SpBMP-9 induced a higher level of pGSK3β (Ser9) than BMP-9 or the CTL, as confirmed by relative fluorescence intensity analysis (p < 0.001 and p < 0.01 respectively). In addition, cells incubated with RA alone or combined with BMP-9 or its derived peptides contained more phosphorylated GSK3β on Ser9 than did cells stimulated without RA (p < 0.001).

## Discussion

The search for an effective treatment for AD has been, and continues to be, intense. The disease has three main hallmarks: cholinergic system dysfunction^4^, beta amyloid plaque accumulation^5^ and protein Tau hyperphosphorylation^6, 7^. Several studies have indicated that GFs may be potent therapeutic agents, particularly those of the TGF-β family, as they act on more than one hallmark simultaneously (8–14). A recent study showed that the plasma of 99 AD patients had a deficient TGFβ/GDF/BMP transcriptome, proteasome and signalling. This study also indicated that the TGFβ member, growth differentiation factor 3 (GDF3), could be a potent therapeutic agent against AD since it plays a role in adult neurogenesis^46^. BMPs are also part of the TGFβ family. They influence brain development and neurogenesis^8, 17–19^ and so could have a great impact on brain degenerative diseases, particularly AD. A recent study found that BMP-4 expression increases in mice dentate gyrus as they age, leading to an increase of canonical Smad signalling and a decrease in BMP inhibitor Noggin which were associated with a decline in cognitive functions^47^. It was also shown in mice that BMP signalling regulates the depressive behavior^48^. Nonetheless, BMPs are classified into several subfamilies depending on their sequence homologies^21^. Other BMPs such as BMP-9, which are not part of BMP-4 subfamily, have shown promising effect in the context of AD^11–13^. Lopez-Coviella *et al*. demonstrated that BMP-9 was the most efficient of the BMPs tested (BMP-2, -4, -6, -7, -9 and -12) at increasing the acetylcholine in septal neurons as well as their expression of neuron differentiation markers such as βIII-tubulin, ChAT and VAchT^11^.

However, proteins do not readily cross the blood brain barrier, most are too big. We therefore used two small peptides (23 residues) derived from the knuckle epitope of BMP-9, corresponding to the sequence recognized by type II BMP receptor (BMPRII), to overcome the size limitation and reduce their cost, since short peptides of similar size (980 Da–6.5 kDa) can be delivered intranasally and successfully cross the blood brain barrier^49^. Our peptides sequences were based on the previous work of Saito *et al*.^29, 50^ on BMP-2. They showed that peptides derived from the knuckle epitope of BMP-2 targeting Type II BMP receptor (BMPRII) could activate the canonical Smad signalling pathway in the context of bone repair in a similar manner as the whole protein. They have also demonstrated that the peptide could interact with both Type I and Type II BMP receptors.

We therefore show that pBMP-9 and SpBMP-9 with or without RA were able to activate the canonical Smad1/5 pathway in SH-SY5Y cells, a well-used *in vitro* model to study the effect of drugs against AD^33, 34^. The SH-SY5Y cells expressed both BMPRIb and BMPRII receptors^19^. The receptor BMPRII is also present in all the brain regions associated with memory and task learning (hippocampus and cortex)^51^. We have already shown in previous papers that pBMP-9 and SpBMP-9 were also able to activate the canonical Smad signalling pathway in a similar way than BMP-9 in murine mesenchymal stem cells C3H10T1/2^31^ and murine preosteoblasts MC3T3-E1^31, 32^. However, we found that the Smad1/5 activation and their nuclear translocation in SH-SY5Y cells in serum free medium appear lower with pBMP-9 or SpBMP-9 compared to BMP-9. Hegarty *et al*. have shown that the Smad pathway activation in SH-SY5Y cells cultured in medium supplemented with 10% (v/v) foetal calf serum is required for BMP-2 induced neurite outgrowth^19^. They also recently found that dynamin dependent endocytosis of BMP receptor complexes allows the maximal effect of BMP-2 (200 ng/mL) on neurite outgrowth in SH-SY5Y cells cultured in medium containing serum^52^. We confirmed that pBMP-9 and SpBMP-9 at concentrations in the physiological range of BMP-9 (~20 ng/mL) in human^53^ stimulate the neurite outgrowth in SH-SY5Y cells in serum free medium. However, despite a strong Smad1/5 activation and their nuclear translocation, SH-SY5Y cells stimulated by BMP-9 in serum-free medium were not able to develop significant neurite outgrowth within 5d in comparison to the control. The addition of RA is required to observe some increase in BMP-9 induced neurite outgrowth. Furthermore, BMP-9 at 1 nM drastically alters the morphology of SH-SY5Y cells, making them more neuroblastoma-like. Further studies are therefore required to better understand by which signalling pathways pBMP-9 and SpBMP-9 induced neurite outgrowth in SH-SY5Y cells.

In addition, we verified that both BMP-9 derived peptides did not significantly decrease the cell number after incubation for 3d and 5d with or without RA. BMP-9 and its derived peptides with RA had also an effect on the metabolic activity of cells, which is unlikely caused by cell proliferation. However, other peptides, such as C-peptide^54^ or other GFs like IGF-1, IGF-2^55^ and NGF^56^ have also shown to have simultaneous effects on the proliferation and neurite outgrowth of SH-SY5Y cells cultivated in serum-free or poor-serum conditions. We also found that RA alone had no significant effect on the metabolic activity of the SH-SY5Y cells. Some studies observed that RA can stop cell proliferation and promote differentiation in SH-SY5Y cells^57–59^. In contrast, it was also shown that differentiation of SH-SY5Y cells induced by sequential treatment using RA and brain-derived neurotrophic factor (BDNF) lead to an increase in ATP level and plasma membrane activity as well as an increase in metabolic need^60^. The impact of the increased metabolism induced by BMP-9 and its derived peptides in the presence of RA on subsequent SH-SY5Y cell behavior remains unclear.

We then confirmed that pBMP-9 and SpBMP-9 stimulate more the differentiation markers expression (MAP-2, NeuN and NSE) than the whole protein BMP-9. We also observed an increase in MAP-2 expression between 3d and 5d in cells stimulated by BMP-9 alone in serum free medium, despite no significant increase in neurite outgrowth at 5d compared to the control. Significant neurite outgrowth in the presence of BMP-9 may take longer time to occur and/or other factors may be required to promote neurite outgrowth^61–63^. Other peptides derived from GFs, including neurotrophic factors such as NGF, stimulate neuronal differentiation^64, 65^. Colangelo *et al*. have shown that 2 small peptides derived from functional sections of NGF can induce neuronal differentiation of PC12 cells just like the whole protein^64^. We also found that RA alone favors neurite outgrowth and increased expression of neuronal markers. RA is known to induce SH-SY5Y differentiation^36, 66–68^. Lopes *et al*. showed that SH-SY5Y cells incubated for 4 and 10d in low (1% v/v) serum medium plus RA contained more terminal differentiation proteins like NSE and NeuN than did cells incubated without RA^66^. RA also enhances the neuronal differentiation induced by pBMP-9 and SpBMP-9. The positive effect of RA plus GFs has been observed using sequential treatment^58, 69^. Several studies have shown that SH-SY5Y cells stimulated with RA plus BDNF had increased neurite outgrowth and length synergistically and more early and late neuronal differentiation markers like MAP-2 and NSE^58, 69^.

Cholinergic system dysfunction is a major hallmark of AD and BMP-9 is known to promote the cholinergic differentiation of neuronal cells^11^. We have shown that both pBMP-9 and SpBMP-9 enhance the cholinergic phenotype of SH-SY5Y cells, with SpBMP-9 being the more efficient. The difference in the effects of SpBMP-9 and pBMP-9 might be due to small differences in their sequences. The two Cysteines in pBMP-9 are replaced by two Serines in SpBMP-9, based on the work of Saito *et al*. on BMP-2^50^. They demonstrated that this small modification on BMP-2 derived peptide increased its affinity for type II BMP receptors, so increasing the osteogenic activity^50^. We find that 0.1 nM SpBMP-9 stimulates the synthesis of ChAT, while SpBMP-9 at 1 nM significantly increases (60%) intracellular Ach. It also enhances the production of VAchT and its distribution within the cell processes, indicating the accumulation of Ach. Adding RA to the culture system also enhances AchE activity and VAchT expression in all our experimental conditions. It is already known that RA directs SH-SY5Y cells towards the cholinergic phenotype and increases their AchE activity^70, 71^.

We have also demonstrated that pBMP-9 and SpBMP-9 act on another AD hallmark by preventing the activation of GSK3β, a Tau kinase. pBMP-9 and SpBMP-9 (0.1 nM) increase the activation of the PI3K/Akt pathway at 2 h and inactivate GSK3β in a time dependent manner from time 0 to 4 h. Activation of the PI3K/Akt pathway in the central nervous system regulates GSK3β and plays an important role in neuron polarity^72^. GSK3β is the most studied Tau kinase since it can be implicated in Tauopathies such as AD^44, 45^. Our immunolabelling studies indicate that 0.1 nM BMP-9 alone had no effect on PI3K/Akt at 2 h compared to the control, which agrees well with its transient effect on GSK3β deactivation via phosphorylation at Ser9. This observation also confirms our published results showing that the level of pGSK3β (Ser9) in SH-SY5Y cells incubated with 1 nM BMP-9 alone gradually decreases from time 0 to 4 h^8^. In contrast, BMP-9 (1 nM) plus RA can maintain the phosphorylation of GSK3β at Ser9 for over 4 h^8^. Indeed, the present study also revealed that RA alone or combined with BMP-9 or its derived peptides can activate the PI3K/Akt/GSK3β pathway. Cheung *et al*. found that SH-SY5Y cells incubated with RA for 7d contained more phosphorylated Akt than the control^36^. RA also protects SH-SY5Y cells against proteasome inhibition-associated cell death via the PI3K/Akt pathway, which is common in AD^57^. Since several studies have demonstrated that inhibiting GSK3β prevents Tau hyperphosphorylation at many sites^33, 73^, pBMP-9 and SpBMP-9, with or without RA, might be effective anti-hyperphosphorylated-Tau agents that could also protect cells against oxidative stress programmed cell death.

RA and other vitamin A derivatives have already been proposed for AD therapy, as has BMP-9. Our present results indicate that two peptides derived from the knuckle epitope of BMP-9, pBMP-9 and SpBMP-9, stimulate the differentiation of SH-SY5Y cells *in vitro* better than the whole protein. SpBMP-9 or pBMP-9 induced terminal neuron differentiation, promote cholinergic differentiation and inactivate GSK3β. Thus, BMP-9-derived peptides, especially SpBMP-9, alone or combined with RA are worth our attention since they act on several AD hallmarks simultaneously. Their small size should make them easier to deliver to the brain tissue. Further *in vitro* with neuronal stem cells and *in vivo* studies in appropriate mouse model are needed to confirm the effectiveness of these promising peptides.

## Materials and Methods

### Material

Recombinant carrier-free human BMP-9 was purchased from R&D system Inc. (Minneapolis, MN, USA), pBMP-9 and SpBMP-9 were from EZBiolab (Carmel, IN, USA). Retinoic acid (RA) (Sigma Aldrich, ON, CA) was dissolved in pure ethanol, and kept frozen in the dark.

### Cell culture

SH-SY5Y human neuroblastoma cells (ATCC® CRL-2266^TM^) were grown on treated polystyrene plates in DMEM/F12 (1:1) Glutamax (Gibco®, Grand Island, NY, USA) containing 1% (v/v) streptomycin (100 mg/mL) and penicillin (100 U/mL) plus 10% (v/v) heat-inactivated fetal bovine serum (FBS) (Wisent, St-Jerome, QC, CA) at 37 °C under a humidified 5% CO_2_/air environment. Cells were passaged at 80% confluence using trypsin-EDTA (0.25%) (Gibco®, Grand Island, NY, USA). The trypsin was neutralized with 10% (v/v) FBS and cells were collected by centrifugation. In all experiments, cells were stimulated in serum-free culture medium with or without 10 µM retinoic acid (RA). The ethanol in which the RA was dissolved never exceeded 0.1% (v/v) of the medium volume.

### Viability assays

SH-SY5Y cells were grown to 80% confluence on 24-well plates, washed with sterile phosphate-buffered saline (PBS) and incubated in serum-free culture medium with equimolar concentrations 0, 0.1 or 1 nM of BMP-9, pBMP-9 or SpBMP-9 with or without 10 µM RA for 1d, 3d and 5d. Viability was assessed using MTS CellTiter 96® Aqueous One Solution Cell Proliferation Assay kit following the manufacturer’s instruction (Promega, Madison, WI, USA). Supernatants were collected after incubation with the MTS reagent and absorbance at 490 nm was measured using a Biotek Synergy HT (Biotek® Instruments Inc., Winosski, VT, USA). Tests were performed in DMEM without phenol red (Gibco®, Grand Island, NY, USA) to avoid any spectrophotometric bias. Results are representative of at least two independent experiments performed in duplicate.

### Morphology analysis

SH-SY5Y cells were grown to 80% confluence on 6-well plates, washed with sterile PBS and incubated with equimolar concentrations (0, 0.1 or 1 nM) of BMP-9, pBMP-9 and SpBMP-9 in serum-free medium with or without 10 µM RA for 5d. 5–10 representative pictures (magnification: 20X) were taken using an Eclipse TE200-S microscope coupled to a CCD camera (Zeiss AxioCam MRm, Car Zeiss, DE). Pictures were smoothed with a Gaussian filter to remove noise, with a Laplace high-pass filter and then with a median filter to detect and enhance neurites using an image analysis app (MatLab *Image Processing Toolbox;* MatLab 2007b, Mathworks, USA). The resulting images showing the neurite edges (blue lines) were superimposed on the original phase-contrast images. Original pictures were shrunk to fit within the figure panel. Each experiment was performed at least three times in duplicate.

### Measurement of neurite length

Mean neurite lengths were determined from the normal size high resolution neurite-enhanced pictures taken for morphology analyses (20x magnification) using an image analysis app (Matlab *Image Processing Toolbox;* MatLab 2007b, Mathworks, USA). Briefly, neurite length was defined as the Euclidean distance in pixels or a combination of Euclidean distances between the cell body and the end of a neurite. The longest path was always chosen when a neurite had several branches. The length in pixels was then calibrated based on the length bar provided by the microscope image treatment software (AxioVision LE, Carl Zeiss, DE). For each experimental condition, 15 to 30 neurites were measured for each picture (4 × 10^−3^ cm²) and at least 5 pictures were analyzed per condition performed. Those steps were done three independent times in duplicate. The mean neurite length for each picture analyzed within a replicate was weighted by the number of measurement taken. For each independent experiment, the mean was calculated as the weighted mean (relative to the inverse of the variance) of each replicate. Finally, the overall mean was calculated as the weighted mean (relative to the inverse of the variance) of each of the three independent experiments. Overall, a total of at least 300 neurite length measurements were obtained for each experimental condition.

### Western blotting and densitometric analysis of signalling protein and neuronal differentiation marker bands

Cells were grown to 80% confluence in 100 mm diameter tissue culture wells, washed with sterile PBS and stimulated with 0.1 nM BMP-9, pBMP-9 or SpBMP-9 in serum-free medium for 3 - 5d for neuronal differentiation markers and between 0 to 240 min for phosphorylated and total Smad 1/5/8 or phosphorylated GSK3β (Ser9). Treated cells were washed with cold sterile PBS containing 1 mM orthovanadate (Sigma) and lysed with 50 mM Tris-HCl (pH 7.4) containing 1 mM orthovanadate (Sigma), 0.1% (v/v) SDS and a complete mini-protease inhibitor cocktail (Roche Diagnostics, Indianapolis, IN, USA). Equal amounts of protein (15 μg) were separated by SDS-PAGE and transferred to polyvinylidene fluoride (PVDF) membranes. Transferred proteins were incubated overnight at 4 °C with primary antibodies against βactin diluted 1:2,000 (Sigma), MAP-2 diluted 1:1,000 (Cell Signalling, MA, USA), phosphorylated and total Smad diluted 1:1,000 (Cell Signalling, MA, USA) or phosphorylated GSK3β (Ser9) diluted 1:1,000 (Cell Signalling, MA, USA). Membranes were washed with PBS containing 0.1% (v/v) Tween 20 and incubated with anti-rabbit secondary antibodies (1:10,000) or anti-mouse secondary antibodies (1:10,000) coupled to horseradish peroxidase. Immunoreactive bands were visualized by chemiluminescence (ECL + Plus TM, GE Healthcare, Buckinghamshire, UK) and exposed to X-ray films (Thermo scientific, Rockford, Il, USA). Films were then digitalized using a high resolution scanner (Canon LidE 120, resolution: 600 × 600 DPI) and the densities of bands determined with an image analysis app (Matlab *Image Processing Toolbox*; MatLab 2007b, Mathworks, USA). Briefly, the intensities of all pixels in a user-defined region of interest (ROI) corresponding to a protein band were summed. The average background, the pixel intensities in an area near the ROI, was then subtracted from the ROI intensity to obtain the integrated density value (IDV). The same ROI dimensions were used for all bands in all experiments. The IDV of the bands of interest were normalized to their corresponding βactin bands, and then to the control without RA in order to account for the variability of each independent experiment. Results are representative of at least 2 independent experiments.

### Immunolabelling of neuron differentiation markers, Smad, GSK3β and Akt

SH-SY5Y cells grown to 80% confluence on sterile microscope coverslips were washed with PBS and incubated with equimolar concentrations (0.1 or 1 nM) of BMP-9, pBMP-9 or SpBMP-9 with and without 10 µM RA for 5d for neuron differentiation markers and with 0.1 nM BMP-9, pBMP-9 or SpBMP-9 +/− 10 μM RA for 2 h (Akt) or 4 h (GSK3β). Treated cells were fixed by immersion in 3% (w/v) paraformaldehyde (Sigma) for 15 min at room temperature and permeabilized by immersion in 0.5% (v/v) Triton X-100 (Sigma) for 5 min. Non-specific sites were blocked by incubation with 1–3% (m/v) bovine serum albumin at 37 °C for 30 min. The coverslips/cells were then incubated at 37 °C for 30 min with primary antibodies: phosphorylated Smad1/5: anti-phospho-Smad 1/5 (Ser463/465) (41D10) rabbit mAb diluted 1:50 (Cell Signalling, MA, USA), βIII-tubulin: anti- βIII-tubulin IgG clone AA2 + AlexaFluor 488 conjugated diluted 1:250 (EMD Millipore, Etobicoke, ON, CA), microtubule-associated protein 2 (MAP-2): anti-MAP2 monoclonal IgG clone AP20 diluted 1:120 (EMD Millipore, Etobicoke, ON, CA), neuron-specific antigen (NeuN): anti-NeuN monoclonal IgG diluted 1:50 (EMD Millipore, Etobicoke, ON, CA), NSE: anti-NSE monoclonal IgG clone 5E2 diluted 1:200 (EMD Millipore, Etobicoke, ON, CA), ChAT: goat anti-ChAT IgG diluted 1:100 (EMD Millipore, Etobicoke, ON, CA), VAchT: anti-VAchT polyclonal IgG diluted 1:100 (EMD Millipore, Etobicoke, ON, CA), Anti-phosphorylated Akt (Thr308) diluted 1:100 (Cell Signalling, MA, USA) or anti-phosphorylated GSK3β (Ser9) diluted 1:100 (Cell Signalling, MA, USA). The coverslips/cells were then washed with PBS and incubated (37 °C for 30 min) with secondary antibodies: anti-mouse polyclonal IgG conjugated to AlexaFluor 488 diluted 1:400 (Life Technologies, Carlsbad, CA, USA), anti-rabbit polyclonal IgG conjugated to FITC diluted 1:100 (Sigma), anti-rabbit polyclonal IgG conjugated to Cy3 diluted 1:100 (Sigma) or anti-goat polyclonal IgG conjugated to FITC diluted 1:200 (Sigma). Cell nuclei and actin cytoskeleton were counterstained with Hoechst 33342 (5 µg/mL) (Life Technologies, Carlsbad, CA, USA) and phalloidin-Alexa 594 diluted 1:100 (Life Technologies, Carlsbad, CA, USA) respectively. The coverslips/cell were mounted on microscope slides with 1:1 PBS-glycerol solution and at least 10 fluorescence pictures for each color filter per replicate were randomly taken using an Evos FL auto fluorescence microscope (Life Technologies, Carlsbad, CA, USA); magnification: 10X and 40X. Results are representative of at least 2 independent experiments. For each color channel, the same exposure time, digital gain and light intensity were used.

### Measurement of immunofluorescence relative intensity

Immunofluorescence relative intensity quantification was assessed using a pixel-wise image analysis app (Matlab *Image Processing Toolbox;* MatLab 2007b, Mathworks, USA). Briefly, the relative fluorescence intensity (RFI) corresponding to the marker of interest in each picture was calculated as the cumulative sum of the value of each pixel above a pre-determined threshold. The cumulative sum of the given marker of interest of each image was then normalized in respect to the RFI of the staining corresponding to the nucleus following the same procedure. Since the pictures of every independent experiment had the same fluorescent light intensity, acquisition time and exposure for each channel, experimental condition could be compared together. At least 5 to 10 pictures with a magnification of 40X were analyzed per independent experiment for each experimental condition and at least 2 independent experiments were performed. The overall RFU mean (RFI of marker of interest/RFI of the nuclei) was determined as a weighted mean relative to the inverse of the variance of each independent experiment.

### Intracellular acetylcholine and acetylcholinesterase assay

SH-SY5Y cells were grown to 80% confluence on 60-mm petri dishes, washed with sterile PBS and incubated for 3d and 5d with equimolar concentrations (0, 0.1 or 1 nM) of BMP-9, pBMP-9 or SpBMP-9 in serum-free medium with or without 10 µM RA. Incubated cells were washed with cold sterile PBS and lysed at 4 °C with 50 mM Tris-HCl (pH 7.4) containing complete mini-protease inhibitor cocktail (Roche Diagnostics, Indianapolis, IN, USA). Supernatants were collected and protein concentrations, OD at 280 nm, were measured with Biotek Synergy HT with a Take3 micro-volume plate adaptor and Gene5 data analysis software (Biotek® Instruments Inc., Winosski, VT, USA). Acetylcholine (Ach) concentration and acetylcholinesterase (AchE) activities were assayed by fluorescence detection using Amplex® Red Acetylcholine/Acetylcholinesterase Assay Kit (Life Technologies, Carlsbad, CA, USA) following the manufacturer’s instructions. Briefly, Ach was measured by mixing samples with choline oxidase, horseradish peroxidase, Amplex Red® reagent and excess AchE; AchE activity was measured with excess Ach. AchE catalyzes the hydrolysis of Ach to acetate and choline, and choline oxidase then breaks down the choline into betaine aldehyde and oxygen peroxide. The resulting oxygen peroxide is reduced by horseradish peroxidase and this reacts with Amplex Red® to give a fluorochrome. The fluorescence, measured with a spectrophotometer (Safire2, Tecan US inc., NC, USA), is a measure of the AchE activity. The Ach concentrations and AchE activities were normalized to the protein content and then to their respective controls. At least four independent experiments were performed.

### Statistical analysis

Statistical analyses were performed with Excel (Excel 2010^®^). Analyses of variance (ANOVA) used confidence limits of 95% followed by a Tukey post-hoc test to determined significant differences within treatments. Only differences with a p < 0.05 were considered significant.

## Electronic supplementary material

Supplementary data 1
