# Supplementary material for: Peptides derived from the knuckle epitope of BMP-9 induce the cholinergic differentiation and inactivate GSk3beta in human SH-SY5Y neuroblastoma cells
Source: Sci Rep. 2017 Jul 5;7:4695. doi: 10.1038/s41598-017-04835-x (PMC5498665; doi:10.1038/s41598-017-04835-x)
Supplement: Supplementary file 1 — Supplementary data 1 [file 41598_2017_4835_MOESM1_ESM.pdf]

PEPTIDES DERIVED FROM THE KNUCKLE EPITOPE OF BMP-9 INDUCE THE CHOLINERGIC DIFFERENTIATION AND INACTIVATE GSK3BETA IN HUMAN SH-SY5Y NEUROBLASTOMA CELLS

Marc-Antoine Lauzon, Olivier Drevelle, Nathalie Faucheux[1]

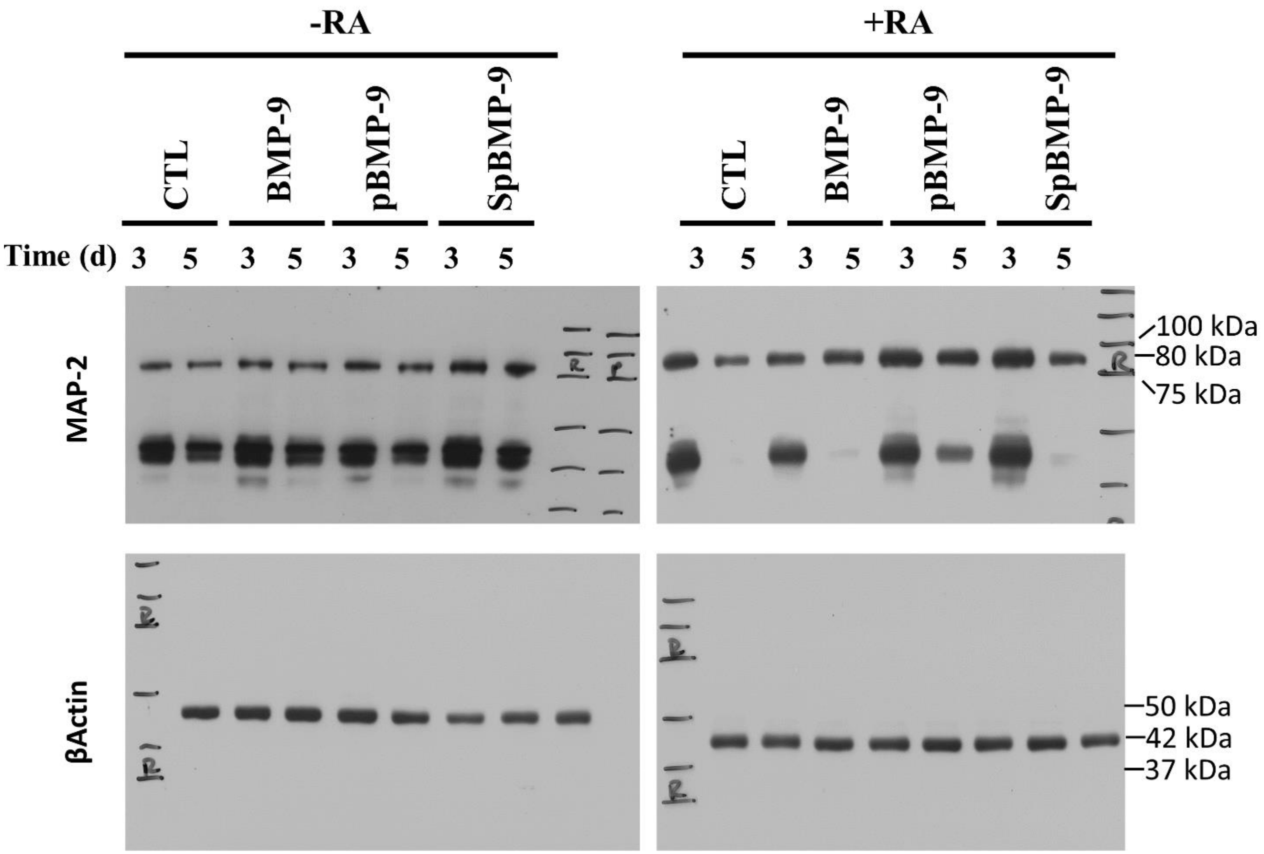

Figure S1: Western blots of MAP-2 and βactin for SH-SY5Y cells stimulated with 0.1 nM BMP-9, pBMP-9 and SpBMP-9 +/- 10 μM RA for 3d and 5d. The 80 kDa band was used since it corresponds to MAP-2 low molecular weight isoform. βActin band, with a MW of 42 kDa was used as a control. Those are the original (non-cropped) blots presented in Figure 4A of the article.

[1] Corresponding author : Tel : +1 819 821 8000 61343, Fax : +1 819 821 7955,
Email : Nathalie.Faucheux@usherbrooke.ca

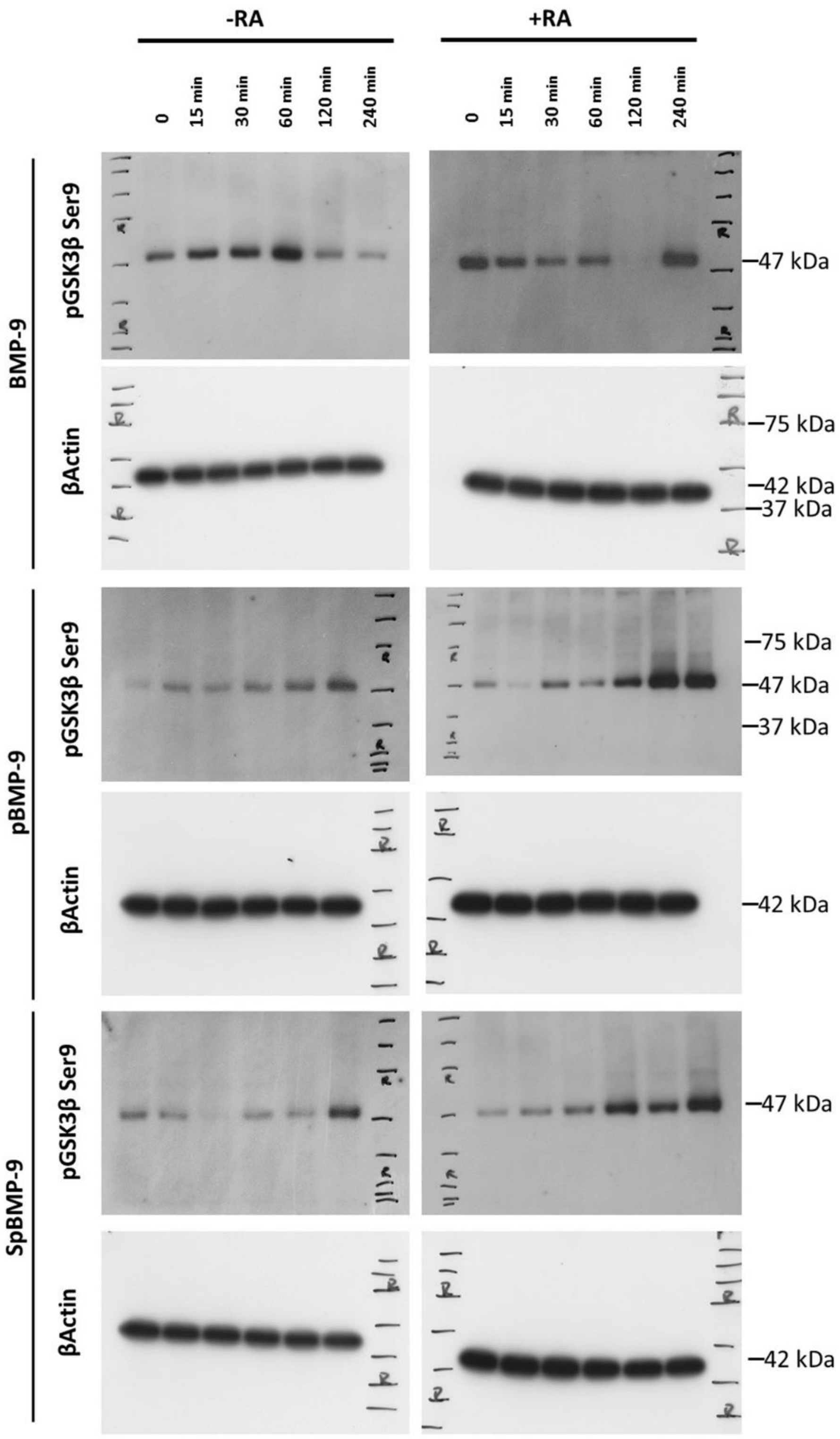

Figure S2: Western blots showing the effect of 0.1 nM BMP-9, pBMP-9 and SpBMP-9 +/- 10 μM RA on the phosphorylation of GSK3β at Ser9 (MW 47 kDa) in SH-SY5Y cells after incubation for 0, 15, 30, 60, 120 and 240 min. βActin (MW of 42 kDa) was used as a control. These pictures are the original (non-cropped) blots presented in Figure 8B of the article.

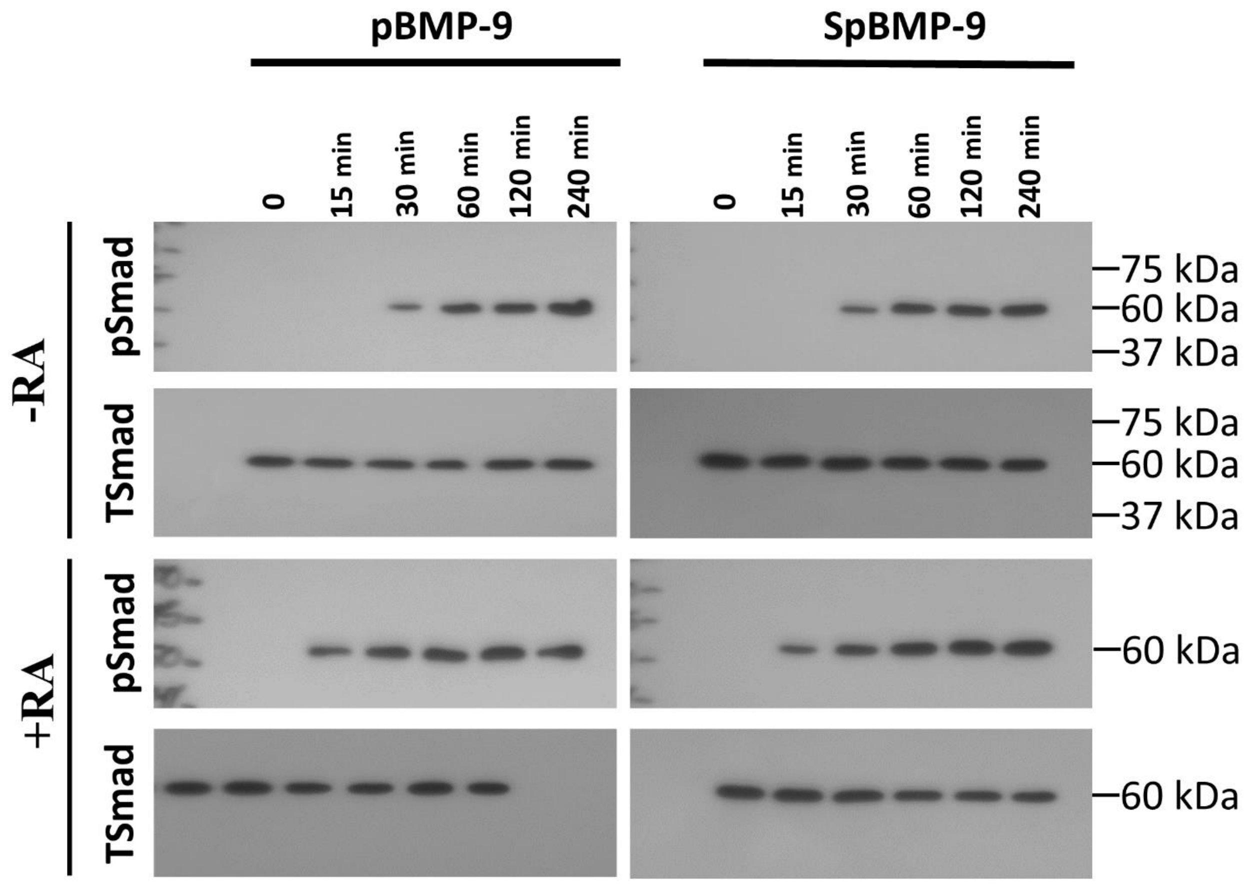

Figure S3: Western blots showing the effect of 1 nM of pBMP-9 or SpBMP-9 +/- 10 µM RA on the phosphorylation of Smad1/5 (pSmad, MW 60 kDa) in SH-SY5Y cells after incubation for 0, 15, 30, 60, 120 and 240 min. Total Smad1/5/8 (TSmad, MW 60 kDa) was used as a control. These pictures are the original (non-cropped) blots presented in Figure 1A of the article.
